# An immune-mediated effect of the antibiotic cefiderocol on LPS-induced acute lung injury

**DOI:** 10.1128/aac.01634-25

**Published:** 2026-02-18

**Authors:** Min Hao, Ying Li, Yingmin Bi, Shuang Liu, Mohan Ju

**Affiliations:** 1 Institute of Antibiotics, Huashan Hospital, Fudan University Shanghai, Shanghai, China; Columbia University Irving Medical Center, New York, New York, USA

**Keywords:** cefiderocol, lipopolysaccharide, acute lung injury, immuno-mediated effect

## Abstract

This study examines the immune-modulatory effects of cefiderocol in a murine model of lipopolysaccharide (LPS)-induced acute lung injury (ALI). Cefiderocol pretreatment significantly reduced lung tissue damage, lowered pro-inflammatory cytokines, and decreased ferroptosis markers. Its protective effect was comparable to that of the ferroptosis inhibitor ferrostatin-1, while cefepime showed limited efficacy. These findings suggest that cefiderocol has an immuno-mediated role in LPS-induced ALI, potentially by modulating ferroptosis and inflammation, warranting further investigation for sepsis-related complications.

**IMPORTANCE:**

This study examines the immune-modulatory effects of cefiderocol in a murine model of LPS-induced acute lung injury (ALI). Cefiderocol pretreatment significantly reduced lung tissue damage, lowered pro-inflammatory cytokines, and decreased ferroptosis markers. Its protective effect was comparable to that of the ferroptosis inhibitor ferrostatin-1, while cefepime showed limited efficacy. These findings suggest that cefiderocol has an immuno-mediated role in LPS-induced ALI, potentially by modulating ferroptosis and inflammation, warranting further investigation for sepsis-related complications.

## INTRODUCTION

Cefiderocol is a novel siderophore cephalosporin used to treat severe sepsis infection caused by multidrug-resistant gram-negative bacteria. The chemical structure of cefiderocol comprises a core β-lactam ring, a pyrrolidinium group that enhances stability against β-lactamases, and a carboxypropanoxyimino group on the C-7 side chain, which facilitates transport across the bacterial outer membrane (1). The defining feature of the cefiderocol structure is the chlorocatechol moiety at the C-3 side chain, which chelates ferric ions (Fe³^+^), promoting active uptake into gram-negative bacteria (2, 3). An *in vitro* study showed that cefiderocol can reduce the inflammatory response triggered by lipopolysaccharide (LPS) in human umbilical vein cells (4). However, the role of cefiderocol in treating LPS-induced acute lung injury (ALI) remains unclear. Therefore, this study aimed to investigate the immune-regulative effects of cefiderocol in a murine model of LPS-induced ALI to assess its therapeutic potential in mitigating inflammation and pulmonary damage associated with sepsis.

All animals were maintained under specific pathogen-free conditions. Thirty-six mice were randomized into six groups (*n* = 6 per group). Group 1 (control) received 0.9% sodium chloride (NaCl) intraperitoneally. Group 2 (cefiderocol) was given a single intraperitoneal dose of cefiderocol (80 mg/kg). Group 3 (LPS) received 10 mg/kg LPS from *Pseudomonas aeruginosa* intraperitoneally, as previously described (5). Group 4 (LPS + cefiderocol) was treated with cefiderocol (80 mg/kg) followed by LPS 2 hours later. Group 5 (LPS + ferrostatin-1 [Fer-1]) was pretreated with the ferroptosis inhibitor ferrostatin-1 (1 mg/kg, tail vein injection) before LPS exposure. Group 6 (LPS + cefepime) received cefepime (100 mg/kg) before LPS administration.

For groups 3 to 6, the murine sepsis score (MSS) was assessed 12 hours after LPS exposure by a blinded observer. The mice were euthanized, and the lung tissues were harvested. The mice in groups 1 and 2 were euthanized 12 hours after NaCl or cefiderocol administration. Lung injury was evaluated by hematoxylin and eosin staining, and the messenger RNA (mRNA) expression levels of inflammatory cytokines and ferroptosis-related markers, including ferrous ion concentration, reactive oxygen species (ROS), malondialdehyde (MDA), and glutathione (GSH), were measured. The experimental design is shown in Fig. S1.

**Fig 1:**
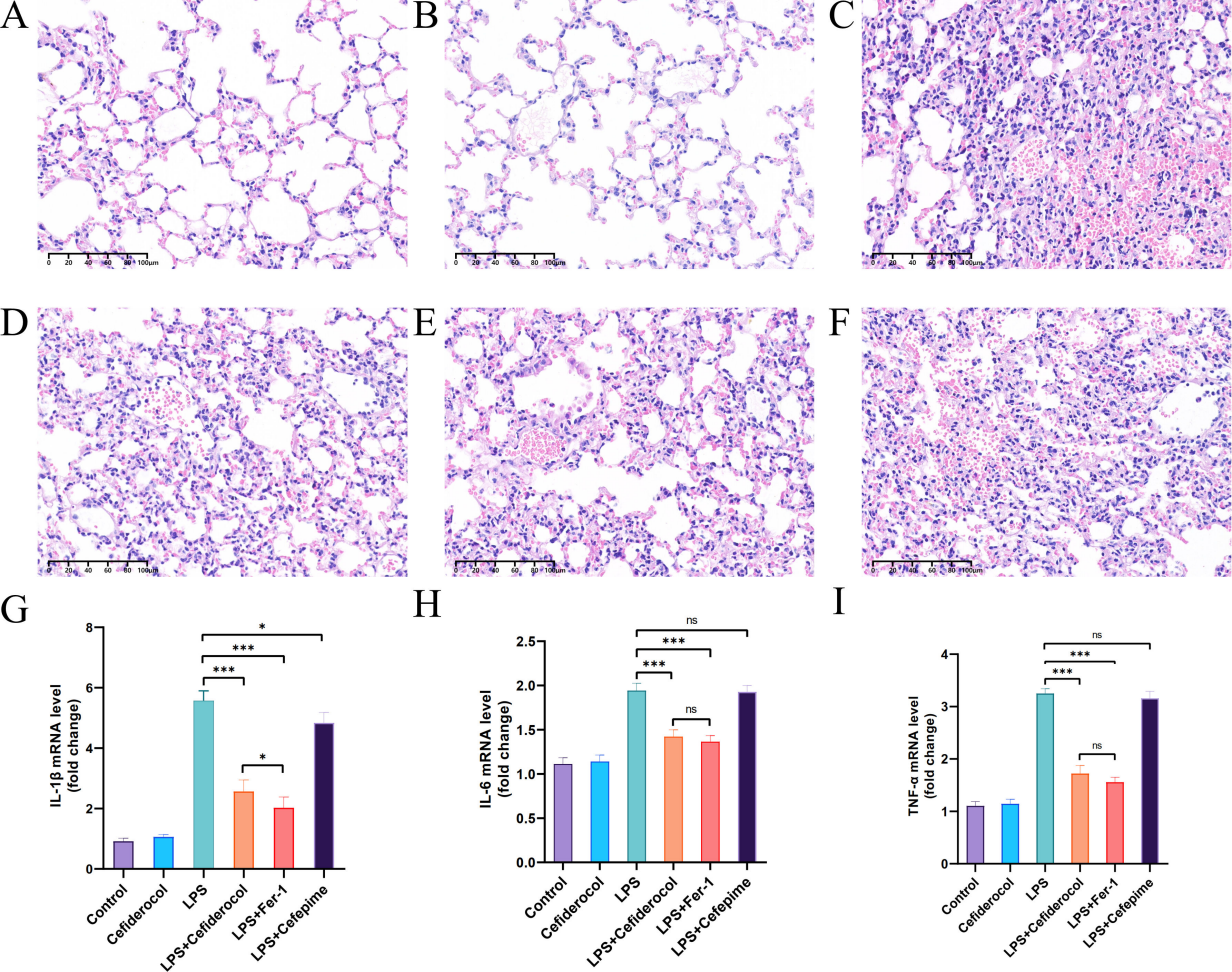
Cefiderocol significantly alleviates LPS-induced ALI *in vivo*. (**A–F** ) Representative images of H&E staining of lung tissue. Morphology was examined using light microscopy (**A**, control group; **B**, cefiderocol group; **C**, LPS group; **D**, LPS+ cefiderocol group; **E**, LPS+ Fer-1 group; **F**, LPS+ cefepime group). (**G–I**) Relative levels of IL-1β, IL-6, and tumor necrosis factor-alpha (TNF-α) mRNAs in murine lung tissue. Data are presented as mean ± SD. *P < 0.05, **P < 0.01, ***P < 0.001.

Compared with the control and cefiderocol-only treated groups (Fig. 1A and B), the LPS-treated mice exhibited marked lung injury, including alveolar damage, inflammatory cell infiltration, hemorrhage, edema, and thickened alveolar septa (Fig. 1C). Co-administration of cefiderocol or Fer-1 significantly reduced LPS-induced tissue damage (Fig. 1D and E), while cefepime had only partial effects (Fig. 1F). LPS also significantly increased the lung mRNA and protein levels of interleukin (IL)-1β, IL-6, and tumor necrosis factor-alpha, which were suppressed by cefiderocol (Fig. 1G through I). The MSS was also elevated by LPS and reduced by cefiderocol treatment as shown in Fig. S2A. The lung injury scores of all groups are illustrated in Fig. S2B. Flow cytometry revealed that LPS increased ferrous ion and ROS levels in lung tissue, which were partially attenuated by cefiderocol (Fig. S3A and B). These findings indicate that cefiderocol mitigates LPS-induced lung injury and sepsis severity in mice.

LPS-induced lung injury is marked by acute inflammation, lipid peroxidation, and impaired antioxidant defenses. MDA is the most significant aldehyde metabolite generated during lipid peroxidation (6). Moreover, ALI caused by oxidative stress can lead to excessive depletion of GSH (7). MDA and GSH levels are widely recognized indicators of ALI (5). Our findings demonstrate that LPS can significantly elevate MDA and reduce GSH levels in mouse lungs. However, these alterations were reversed by cefiderocol treatment (Fig. S3C and D).

Recent studies have demonstrated that LPS induces oxidative damage to host tissues via the production of inflammatory mediators and ROS, which disrupt iron homeostasis (8, 9). Our results show that cefiderocol partially attenuated LPS-induced ALI and tissue inflammation and markedly inhibited ferroptosis. These effects were comparable to similar studies using the ferroptosis inhibitor Fer-1 (10). In contrast, despite its structural similarity, cefepime had no significant effect on LPS-induced lung damage.

Cefiderocol, a novel antibiotic, is effective against drug-resistant gram-negative bacteria, including difficult-to-treat *P. aeruginosa*, carbapenem-resistant *Acinetobacter baumannii*, and carbapenem-resistant *Enterobacterales* (11, 12). The U.S. FDA approved the use of cefiderocol in September 2020 for hospital- and ventilator-associated bacterial pneumonia. Cefiderocol functions as an iron chelator, inhibiting ferroptosis by blocking electron transfer from iron to oxides and reducing the production of ROS. Our results confirmed the immune-protective effect of cefiderocol in LPS-induced ALI. Conversely, cefepime showed no benefit in treating ALI. However, the use of traditional iron chelators, such as deferoxamine and deferasirox, is currently limited by instability, poor patient compliance, and adverse effects (13). In contrast, cefiderocol is FDA approved, well tolerated, and may provide a safer, more convenient option to treat sepsis-related conditions associated with iron dysregulation .

Our study has several limitations that have to be acknowledged. We used intraperitoneal LPS injection rather than cecocolonic puncture, which may not fully replicate the pathophysiology of sepsis. ALI involves damage to multiple cell types, including epithelial, endothelial, and immune cells (14, 15). Different modes of cell death, including apoptosis, pyroptosis, and ferroptosis, are interconnected, with activation of one pathway often affecting the others. This crosstalk amplifies cellular damage and inflammation, thus further promoting the development and progression of ALI (16, 17). We did not examine other cell types or alternative cell death pathways in LPS-induced ALI. In addition, our findings are based on animal models, and further clinical studies are needed to confirm the efficacy of cefiderocol in sepsis-related complications.

Overall, cefiderocol significantly mitigated histopathological alterations and cytokine production, thereby reducing the effects of LPS-mediated ALI in mice. Cefiderocol also enhanced the levels of antioxidants in lung tissues while concurrently reducing LPS-induced ferroptosis. Cefiderocol may offer additional immuno-protective effects in LPS-induced ALI cases. However, further clinical studies are warranted to confirm these findings.

## SUPPLEMENTAL MATERIAL

Fig S1 aac.01634-25-s0001.tif Schematic presentation of experimental animal design.

Fig S2 aac.01634-25-s0002.tif Effects of cefiderocol on MSS and lung histopathological score in LPS-induced kidney injury in mouse.

Fig S3 aac.01634-25-s0003.tif Cefiderocol inhibits ferroptosis in murine lung during sepsis-induced ALI.

Supplemental material aac.01634-25-s0004.docx Captions for Supplemental figures.

ASM does not own the copyrights to Supplemental Material that may be linked to, or accessed through, an article. The authors have granted ASM a non-exclusive, world-wide license to publish the Supplemental Material files. Please contact the corresponding author directly for reuse.
