# Supplementary figures and images for: An immune-mediated effect of the antibiotic cefiderocol on LPS-induced acute lung injury
Source: Antimicrob Agents Chemother. 2026 Feb 18;70(4):e01634-25. doi: 10.1128/aac.01634-25 (PMC13041387; doi:10.1128/aac.01634-25)

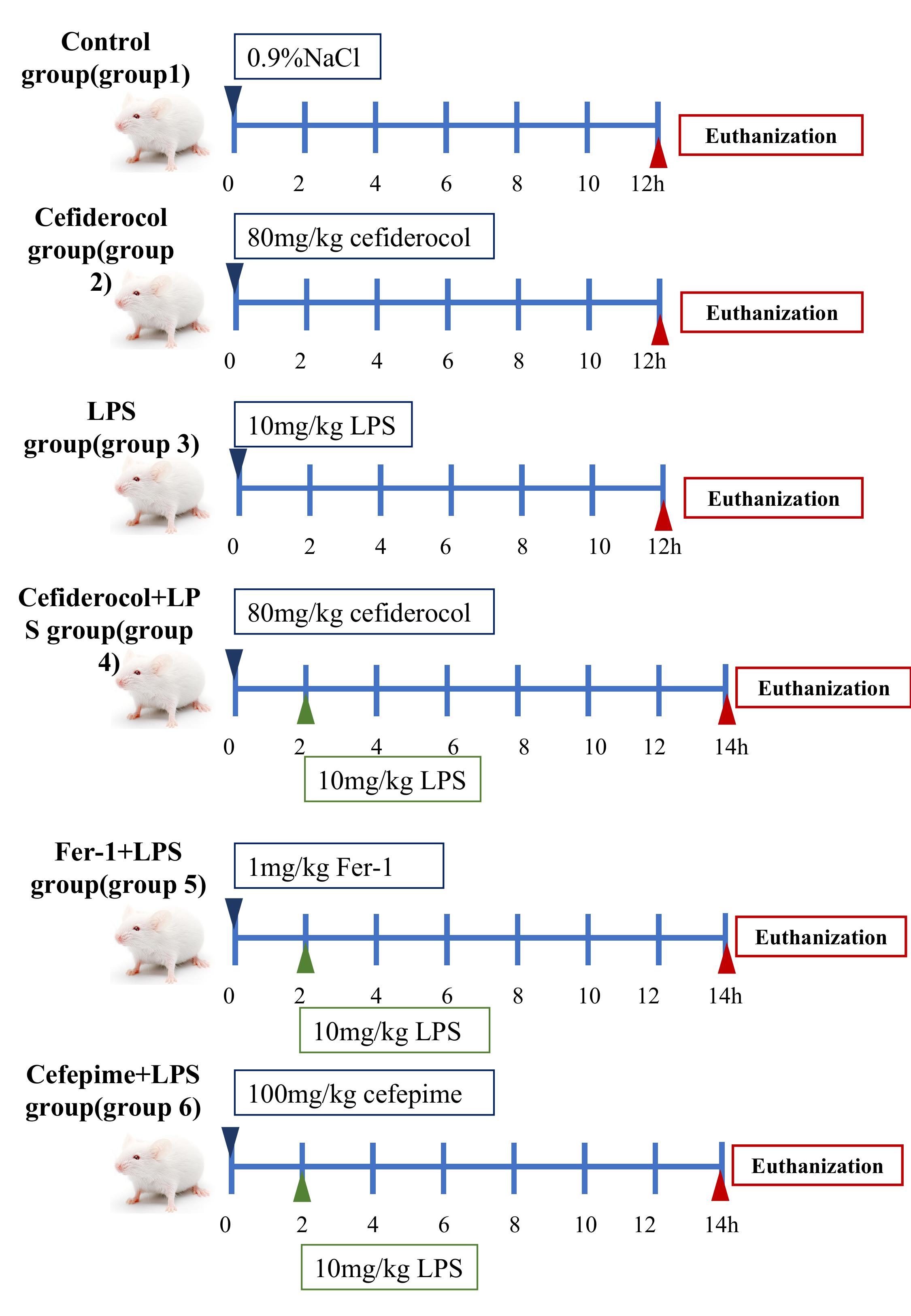

Supplement: Fig S1 — Schematic presentation of experimental animal design. [file aac.01634-25-s0001.tif]

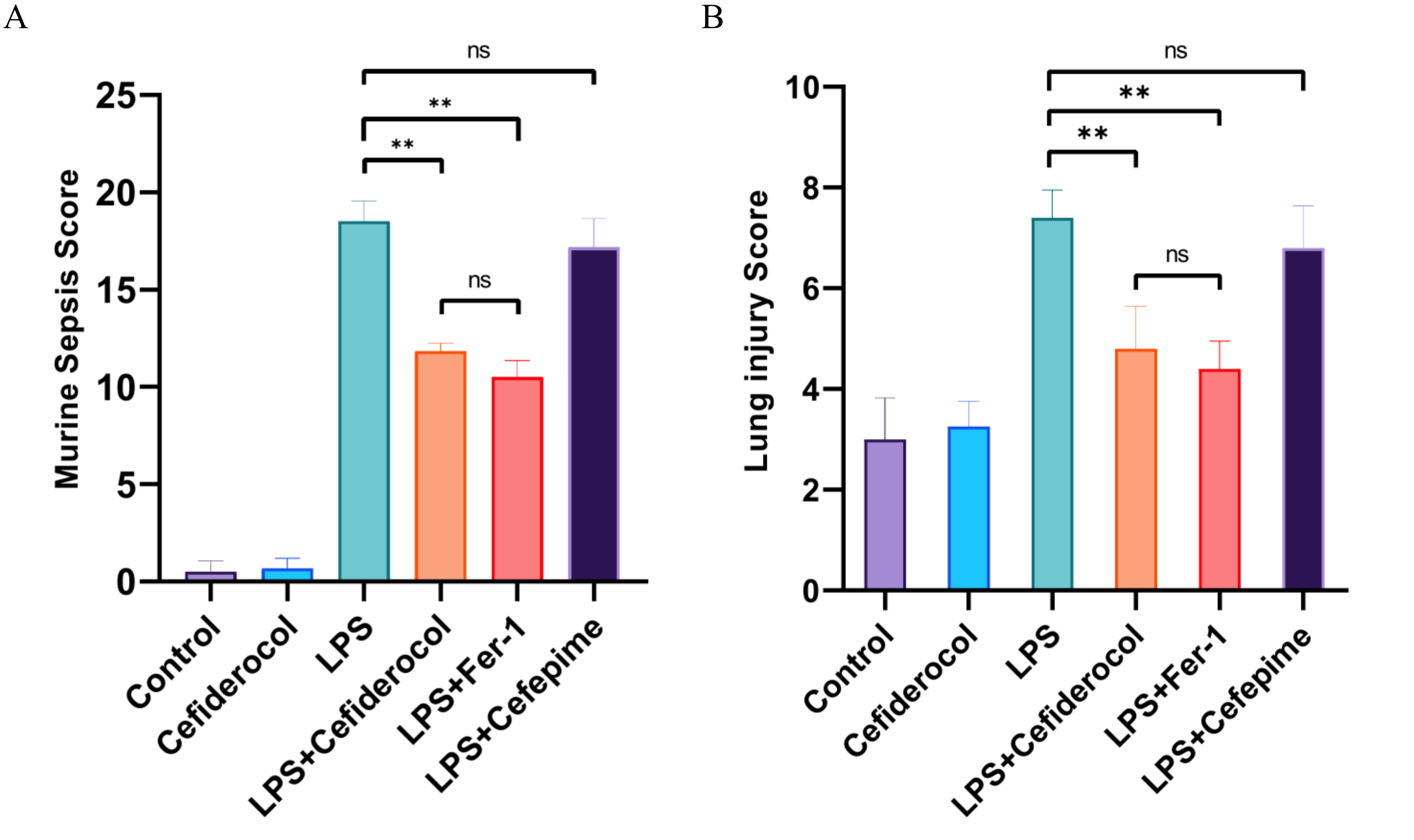

Supplement: Fig S2 — Effects of cefiderocol on MSS and lung histopathological score in LPS-induced kidney injury in mouse. [file aac.01634-25-s0002.tif]

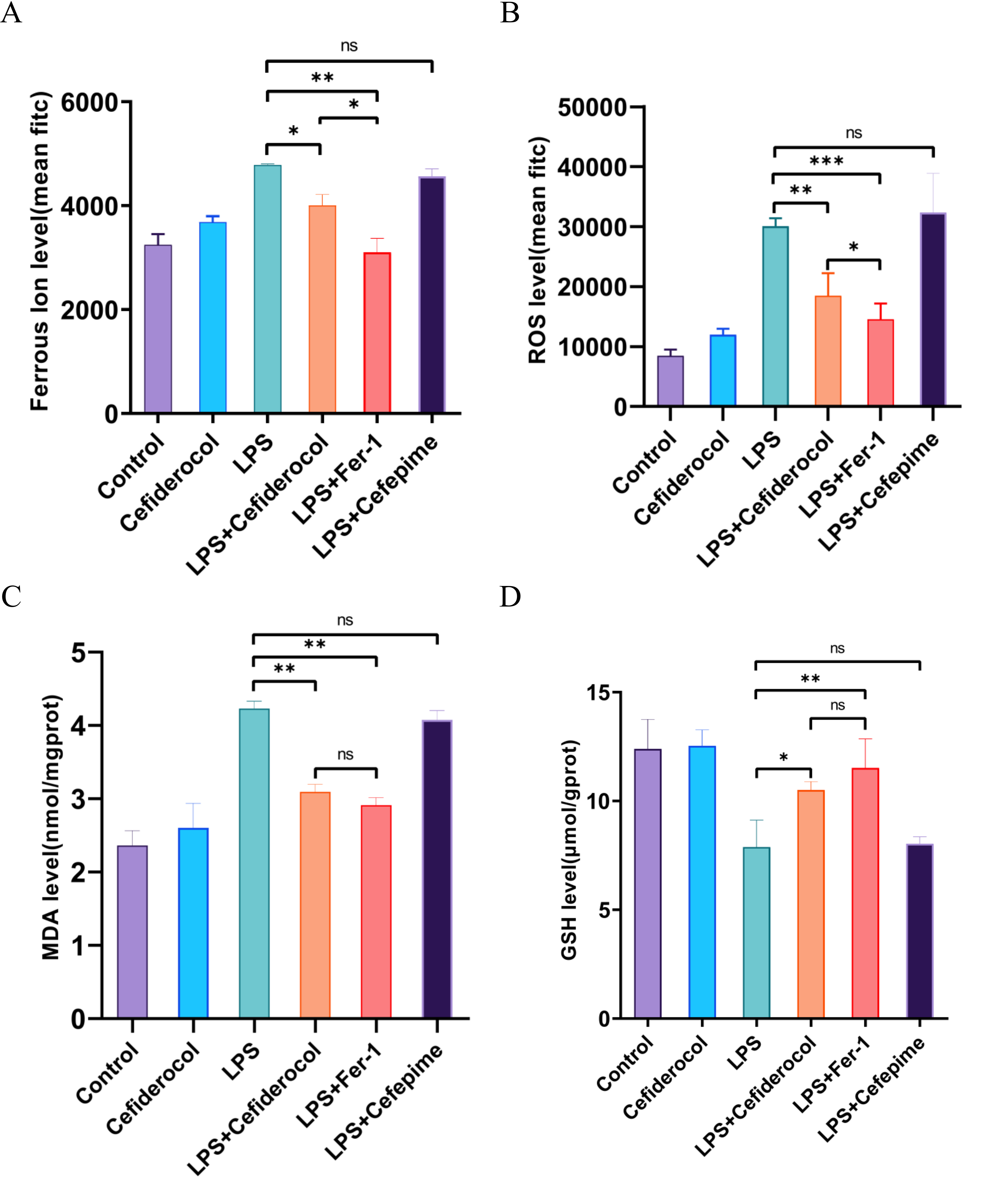

Supplement: Fig S3 — Cefiderocol inhibits ferroptosis in murine lung during sepsis-induced ALI. [file aac.01634-25-s0003.tif]
