# Supplementary material for: An immune-mediated effect of the antibiotic cefiderocol on LPS-induced acute lung injury
Source: Antimicrob Agents Chemother. 2026 Feb 18;70(4):e01634-25. doi: 10.1128/aac.01634-25 (PMC13041387; doi:10.1128/aac.01634-25)
Supplement: Supplemental material — Captions for Supplemental figures. [file aac.01634-25-s0004.docx]

**Figure captions of Supplementary Figure**

**Supplementary Figure.1 Schematic presentation of experimental animal design.**

LPS: Lipopolysaccharide; h: hour.

**Supplementary Figure.2** Effects of cefiderocol on murine sepsis score (MSS) and lung histopathological score in LPS-induced kidney injury in mouse.

A. MSS in groups(n=6) described in panel

B. Semiquantitative histological scores of lung injury in groups(n=6) described in panel (Data are presented as Mean ± SD *p < 0.05, **p < 0.01, ***p < 0.001).

**Supplementary Figure.3** Cefiderocol inhibits ferroptosis in murine lung during sepsis-induced ALI.

A-B. Statistical results of flow cytometry for detecting iron and ROS level in murine lung tissue (n = 6). C-D. MDA and GSH level of murine lung tissue (n = 6). (Data are presented as Mean ± SD *p < 0.05, **p < 0.01, ***p < 0.001).
